# Spatial Pattern and Influencing Factors of Tourism Resources in Northwestern Ethnic Areas in China—A Case Study of Longde County

**DOI:** 10.3390/ijerph192416684

**Published:** 2022-12-12

**Authors:** Shengrui Zhang, Lei Chi, Tongyan Zhang, Yingjie Wang

**Affiliations:** 1 Management College, Ocean University of China, Qingdao 266100, China; 2 Institute of Geographic Sciences and Natural Resources Research, Chinese Academy of Sciences, Beijing 100101, China

**Keywords:** tourism resources, spatial pattern, influencing factors, ethnic areas, China

## Abstract

With the in-depth development of the Western Development Strategy and Rural Revitalization, the tourism industry has become an important economic sector to drive local development in northwestern minority areas of China. This study established a database of tourism resources in Longde county and analyzed the spatial pattern of these tourism resources by using the scale index and kernel density. From the perspective of the natural and social environment, this paper quantitatively discusses the influencing factors of the spatial pattern of tourism resources in the county based on a multiple linear regression model. The results showed that: (1) There were 2049 tourism resources distributed in Longde County, and building and facility resources were the most prevalent. (2) The hierarchical structure of tourism resources in the county showed a “pyramid” distribution, where excellent tourism resources accounted for 22.60% of the total resources. (3) The spatial pattern showed an agglomeration distribution, and the spatial differences of all kinds of resources were significant. The resources were mainly distributed in Chengguan Town and Wenbao Township. (4) Topographic conditions, convenient transportation, population density and water system distribution were important factors that affected the spatial pattern of tourism resources. It was suggested that the government issue corresponding policies to optimize the spatial pattern of tourism resources; balance the relationship between the exploitation of tourism resources, tourism development and local economy, and the social and ecological environment; and promote the sustainable development of tourism in the northwestern ethnic areas in China.

## 1. Introduction

The ethnic areas in northwestern China have a variety of terrain and profound cultural heritage, making it famous for its rich and colorful tourism resources. With the in-depth development of the Western Development Strategy, the ecological civilization strategy and the rural revitalization strategy, the tourism industry has become an important field that stimulates the economic growth of the western region in China [1]. The development of tourism resources was increasingly closely related to national strategies and local policies [2]. General Secretary Xi Jinping pointed out that ecological and cultural resources were the basis for the development of tourism, and historical and cultural heritage should be well used to jointly create tourism products with Silk Road characteristics.

Longde County is located in a minority area of western China, which belongs to the transition zone between arid and semi-arid areas. The geological and geomorphological conditions in the region are complex, and the advantages of its cultural and tourism resources endowment are obvious. However, due to the restrictions of national policies, the regional tourism development level and transportation accessibility, a general survey of the cultural and tourism resources was not carried out. The Ecological Protection and High-Quality Development of the Yellow River Basin policy was proposed as a national strategy in September 2019, where the main goal of the strategy was to protect, inherit and promote the Yellow River culture by laying a foundation for the cultural resource census in Longde County of the Yellow River Basin. In 2021, Ningxia Hui Autonomous Region, Qinghai Province, Xinjiang Kashgar Region, Inner Mongolia Erdos City and other regions successively released the Fourteenth Five-Year Plan for the development of tourism, which all clearly pointed out that the rational and orderly development of tourism resources was the key to promoting high-quality tourism development, and immediately launched the regional cultural and tourism resources census project.

**Figure 1:**
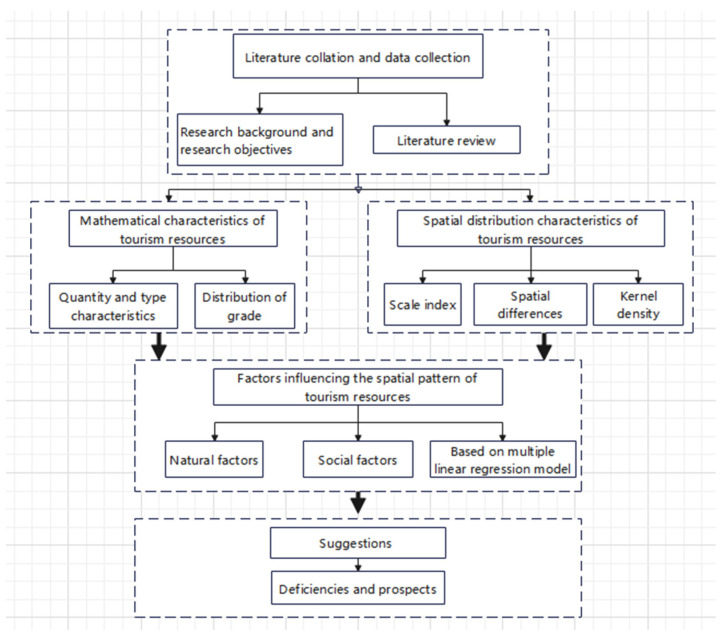
Research framework.

In general, the current work lacks full coverage of the resource census data; while it mainly explores the characteristics and spatial patterns of county-level tourism resources through qualitative descriptions, it often only focuses on a few specific tourism resources. In order to fill this academic gap, the research objectives of this study were mainly based on the National Standard for the “Classification, investigation and evaluation of tourism resources” (GB/T18972-2017), taking Longde County as the study area, where a Longde County tourism resources database was established through a field investigation and a census of other resources. Using quantitative spatial research methods, such as the scale index, kernel density and spatial difference, we analyzed the mathematical characteristics and spatial pattern of tourism resources in Longde County. In addition, a multiple linear regression model was established to quantitatively analyze the influencing factors of the spatial pattern of tourism resources from the perspectives of the natural environment and social environment. Finally, based on the analysis, we put forward a corresponding optimization path and provide a reference for the development of tourism resources in northwestern ethnic areas in China (Figure 1).

Among them, the mathematical and spatial characteristics analysis of regional tourism resources was a prerequisite for discovering the resources, improving the efficiency of resource development and realizing the high-quality development of tourism. This study analyzed the regional spatial pattern and regional differences in tourism resources, clarified the factors that influenced the spatial pattern of tourism resources and quantitatively revealed the influential mechanism, which is helpful to guide the compilation of tourism planning.

## 2. Literature Review

Tourism is a global emerging industry and the development of tourism plays a certain role in promoting the growth of a region’s economy. Balaguer et al. took Spain as a case study and examined the role of tourism in Spain’s long-run economic development [3]. The tourism-led growth hypothesis was confirmed through cointegration and causality testing. Amaghionyeodiwe found that there was a long-term positive relationship between Jamaica’s economic growth and tourism development, and the increase in tourism income tended to have a positive impact on the GDP [4]. Through empirical research, Brida et al. showed that tourism as a factor can promote the growth of the Brazilian economy [5]. Karimi believed that inbound tourism is positively correlated with Malaysia’s economic growth, and in the long run, tourism can become one of the important factors for Malaysia’s economic growth and development [6].

Regional tourism development is a hot topic in academic circles. Iliev’s research conducted on the example of Macedonia showed visible differences in the current stage of the development of tourism between active tourism areas and underdeveloped areas. The unbalanced development of tourism in the country entails the need for planning the development of tourism and the policy of development to reduce regional disparities [7]. At the same time, there are some problems, such as the resource curse effect, inconsistency between tourism development and ecological protection, and asynchronous development of cross-border tourism resources. Zhang et al. found that administrative segmentation causes an imbalance in the development of trans-provincial physical geographic entities, which is mainly manifested by the differences in regional development goals, management models and development timing [8].

As a comprehensive industry, the development of tourism is bound to be affected by many factors, such as regional climate change, as well as economic, political and social factors [9]. In addition, Konstantakopoulou found that the health quality of the host country had a significant impact on international tourism revenue [10]. Zhang et al. believed that transportation accessibility, tourism infrastructure construction, foreign direct investment and the number of employees in the tertiary industry were all important factors that affected the tourism development of Guizhou Province [11]. Many scholars believed that regional tourism resource endowment is also the main factor affecting tourism development [12,13,14]. Therefore, it is necessary to more comprehensively study tourism resources to consider the key factors affecting tourism development. With the continuous development of the global tourism industry, the research content of tourism resources by domestic and foreign scholars is becoming increasingly extensive. The study of the analysis and evaluation of tourism resources in foreign countries precedes in China. As early as the 1950s, relevant researchers began to study the above aspects, but mainly focused on the qualitative analysis and evaluation of resource types [15,16], the evaluation of the value of tourism resources, problems in the development of the tourism industry and countermeasures [17,18,19,20], and the evaluation of the development potential of tourism resources [21]. Among them, taking the northern part of Hainan Province as an example, Lin et al. extracted the factors that affected the performance of regional tourism cooperation from the cognition of relevant stakeholders in the development process of the tourism circle. Finally, they summarized the problems that existed in regional tourism cooperation and put forward suggestions and countermeasures.

In addition, the sustainable development of tourism has gradually become an important topic. Marti et al. conducted quantitative research in the field of sustainable development of tourism and designed a special tool to evaluate the urban sustainability of Spain’s Mediterranean tourist areas. The results of the study found that the development of tourism led to urban sprawl, showing the harmful effects of significant touristic investments [22]. Therefore, a more comprehensive understanding of the extent to which tourism development is sustainable and how we should control the exploitation of tourism resources provides a new idea for subsequent academic research. Chinese domestic scholars conducted a wide range of qualitative research on regional tourism resources at the end of the 20th century [23,24]. In the 21st century, with the development of GIS and other geographic information technologies, research on tourism resources has mainly focused on the analysis of spatial patterns and influencing factors, and traditional qualitative analysis has been gradually replaced by spatial quantitative research [25,26]. The mainstream research methods include spatial association, kernel density analysis, spatial superposition analysis, multiple linear regression and geographical detector methods [13,27,28,29,30,31,32,33]. Zhu et al. believed that altitude, slope and water system distribution were important factors that affected the spatial pattern of religious tourism resources in Qinghai Province. The study area covered the developed areas in eastern China and the less developed areas in western China [34,35,36], as well as China’s land border areas [37]. The research scale was based on the provincial administrative units and municipal administrative units [38]. Resource types included cultural tourism resources [39], sports and leisure tourism resources [40], health tourism resources [41], ecological tourism resources [42], rural tourism resources [43], industrial mining area tourism resources [44], poverty alleviation tourism resources [45] and B&B tourism resources [46]. Among them, Wang et al. found that China’s health tourism destinations were mainly distributed in areas with good environmental quality, high transportation accessibility and a dense potential health population. In general, the existing research on the spatial pattern of tourism resources and its influencing factors showed various forms and rich contents. Most of the relevant research areas in China were concentrated in economically developed areas, while the research content aimed at the characteristics of northwestern ethnic areas was lower.

In summary, the research on the classification, spatial pattern optimization and influencing factors of tourism resources in ethnic minority areas in western China is still insufficient, and the quantitative research results are few. The previous research was mainly conducted at the provincial, prefecture-level city and single scenic spot scales, while studies at the county level were relatively few. Therefore, it is particularly important to carry out a tourism resources survey in Longde County, describe the mathematical characteristics and spatial pattern of the tourism resources, analyze the influencing factors of the spatial distribution pattern and put forward corresponding development suggestions.

## 3. Methods and Data Sources

### 3.1. Study Area

**Figure 2:**
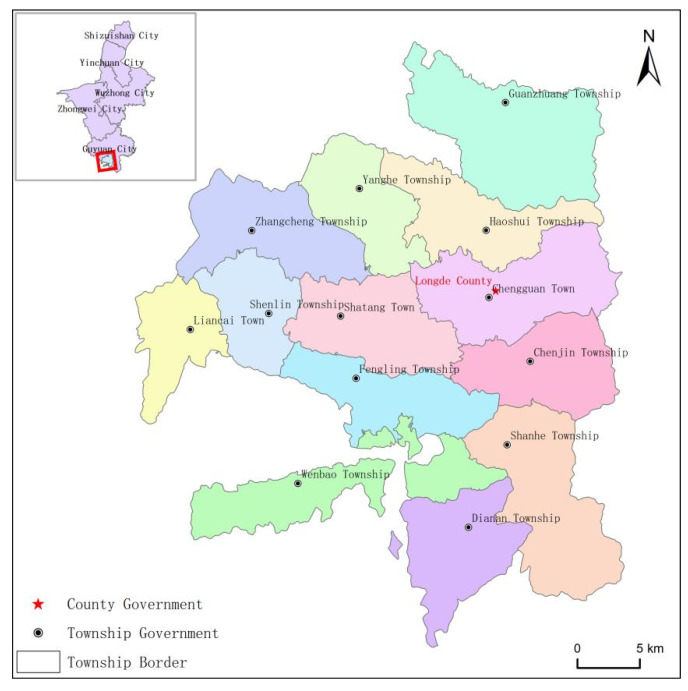
Administrative map of Longde County.

This study took Longde County in the southern part of Ningxia Hui Autonomous Region (Ningan junction) as the research object, which covered 13 townships, 113 administrative villages and 10 communities, such as Chengguan Town and Wenbao Township. The county government was located in Chengguan Town, with a total resident population of 109,400, where ethnic minorities accounted for 16,600 (15.17%) of the total population, with Hui as the main part of the ethnic population (Figure 2). Relying on its unique natural environment and cultural factors, Longde County formed rich tourism resources, especially the multi-ethnic integration of cultural ecology in the northwestern ethnic areas. 

### 3.2. Methods

#### 3.2.1. Scale Index

The scale index was used to calculate the intensity of tourism resources in each township of Longde County, and combined with the visualization analysis in ArcGIS 10.6, it directly reflected the scale difference in the distribution of tourism resources in each township of Longde County. The calculation formula was as follows:

G_i_ = N_i_/S_i_ (1)

where G_i_ represents the scale index of tourism resources in the ith township, N_i_ represents the number of tourism resources in the ith township and S_i_ represents the regional area in the ith township. The larger the G_i_ value, the larger the distribution scale of tourism resources in this region was and the denser the tourism resources were. In contrast, the smaller the G_i_ value, the more dispersed the tourism resources.

#### 3.2.2. Spatial Differences

The coefficient of variation (CV) was used to describe the spatial differences in tourism resources [47], which were calculated as follows:

(2) $$\mathrm{SD}=\sqrt{\frac{1}{n}\sum_{i=1}^{n}{\left(\mathrm{Xi}-\bar{X}\right)}^{2}}$$

(3) $$\mathrm{CV}=\mathrm{SD}/\bar{X}$$

where CV refers to the coefficient of variation, SD is the standard variance, n is the number of administrative units, X_i_ refers to the number of the objects of tourism resources in the ith administrative unit (i = 1, 2, …, n) and $\bar{X}$ refers to the average objects of tourism resources in each administrative unit. If the coefficient of variation was large, it indicated that the spatial difference in tourism resources in Longde County was obvious.

#### 3.2.3. Kernel Density

Kernel density analysis of tourism resources can accurately express the spatial relationship between point elements and line elements, and accurately describe the spatial pattern between the corresponding description elements according to the actual situation to analyze the spatial agglomeration characteristics of tourism resources. The kernel density analysis tool in ArcGIS 10.6 software was used for the calculation and analysis, and the results were visually analyzed. The kernel density calculation equation is as follows:

(4) $$f\left(s\right)\;=\sum_{i=1}^{n}\frac{1}{r}K(\frac{d_{\mathrm{is}}}{r})$$

where f(s) is the kernel density value at s, r refers to the analysis radius, n refers to the number of tourism resource points less than or equal to r at s, d_is_ is the distance from i to s and the K function is the distance decay function [48]. The density value of tourism resources decreases with the increase in distance (d_is_). When the distance is r, the density value of tourism resources is 0.

#### 3.2.4. Multiple Linear Regression Model

The distribution pattern of tourism resources in geographical space is often the result of the action of many factors, and many factors are also interrelated and affected. Therefore, this study used a multiple linear regression model to investigate the comprehensive impact of terrain, water system, transportation and other factors on the spatial pattern of tourism resources. The model used was as follows:

y_a_ = *β*_0_ + *β*_1_*Χ*_1_ + *β*_2_*Χ*_2_ + … + *β*_7_*Χ*_7_ (5)

where y_a_ is the dependent variable; *Χ*_1_, *Χ*_2_,…, *Χ*_7_ are independent variables; and *β*_0_, *β*_1_, *β*_2_,…, *β*_7_ are undetermined parameters.

### 3.3. Data Source

#### 3.3.1. Tourism Resources Data

This study established a tourism resource database using basic geographic data, toponym data and a field investigation. There were 2049 objects of tourism resources included in the tourism resource database, covering 8 main types, 20 sub-types and 70 fundamental types (Table 1), and the field investigation involved 1920 objects. The tourism resource database included the name, basic type, accessibility, development status, longitude and latitude where the township was located, data source and quality level.

#### 3.3.2. Other Basic Data

The data on county administrative boundaries and township administrative boundaries used in this study were obtained from the Data Center for Resources and Environmental Sciences, Chinese Academy of Sciences. The data on main traffic roads and water systems were obtained from the National Basic Geographic Information System database. The DEM data were gathered from the National Basic Geographic Information System database at a resolution of 500 m. The tourism economic data of Longde County was achieved from the Statistical Bulletin of National Economic and Social Development of Longde County.

## 4. Results

### 4.1. Numerical Features of Tourism Resources in Longde County

Due to the different attributes and characteristics of tourism resources, the tourism resources in Longde County were divided into 8 main types, 20 sub-types and 70 fundamental types, such as geological landscapes, water landscapes, biological landscapes, astronomical phenomena and meteorological landscapes, buildings and facilities, ruins and remains, tourism commodities and human activities (Table 1), which were mainly based on the National Standard for the “Classification, investigation and evaluation of tourism resources” (GB/T18972-2017).

In terms of types, the quantities of different types of tourism resources varied greatly. Among the main types, the buildings and facilities type was the largest one, accounting for 47.34%, followed by geological landscapes (29.38%), ruins and remains (14.15%), water landscapes (4.93%), tourism commodities (2.20%), human activities (0.89%), and astronomical phenomena and meteorological landscapes (0.34%, Table 1). Among the sub-types, the natural landscape complex had the largest number (583), followed by the cultural landscape complex (509), practical buildings and facilities (360), material cultural remains (269) and landscape architecture (101). Among the fundamental types, hill landscapes were the most common, with 560 single resources, accounting for 27.33% of the total resources, followed by religious and sacrificial sites, architectural relics and embankments.

**Figure 3:**
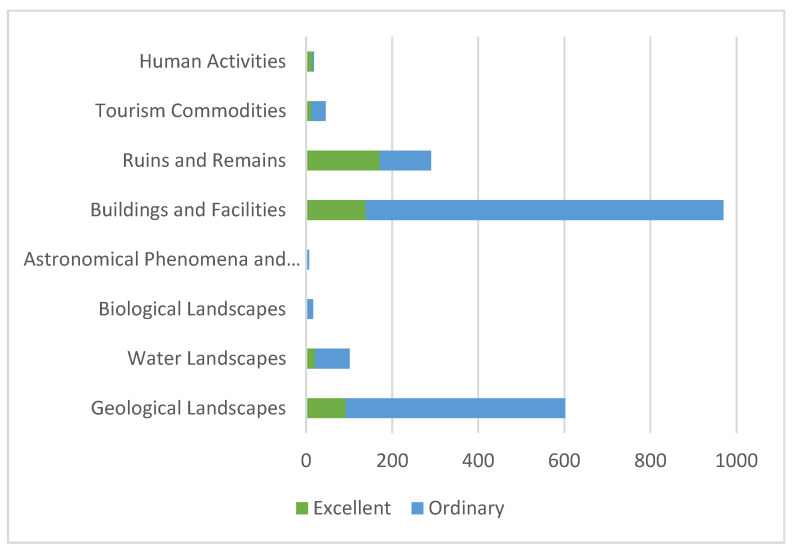
Distribution of different levels of tourism resources according to the main types.

The tourism resources in Longde County showed a pyramidal distribution in terms of the resource grades. The higher the grade, the fewer the number of resources. According to the National Standard for the “Classification, investigation and evaluation of tourism resources” (GB/T18972-2017), there were 463 excellent tourism resources comprising grades 3–5), which was only 22.60% of the total number of resources. There were 1586 ordinary tourism resources (comprising grades 1–2), which constituted the main body of tourism resources in Longde County (Table 2). Among the tourism resources, there were only 16 grade-5 tourism resources, and the buildings and facilities had the best performance, with 9 grade-5 tourism resources. Among the excellent tourism resources, the number of ruins and remains was the largest, with 171 resources. Among the ordinary tourism resources, the buildings and facilities category was the most common, and the number of human activities and biological landscapes was the least with only three objects (Figure 3).

### 4.2. Spatial Pattern of Tourism Resources in Longde County

#### 4.2.1. Scale Index Analysis

**Figure 4:**
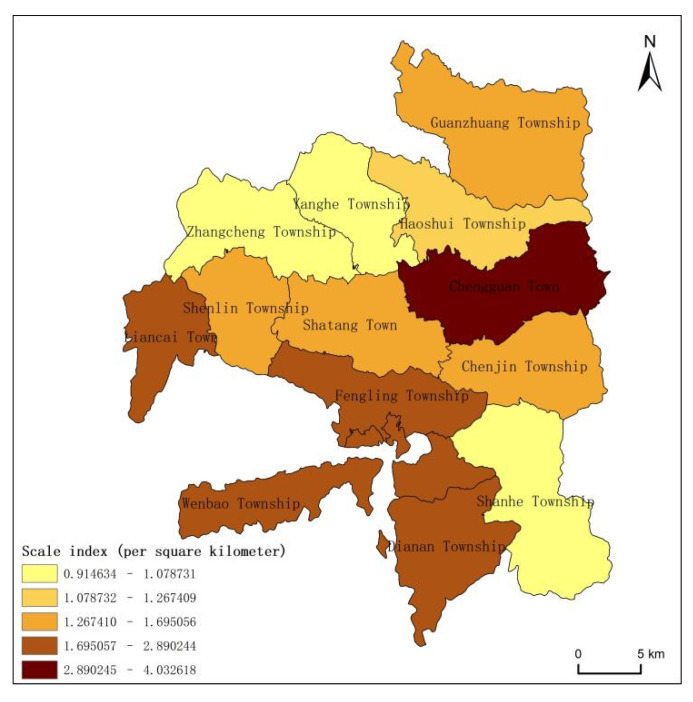
Density distribution of tourism resources in Longde County.

Longde County was rich in tourism resources, but due to the different geographical locations and cultural factors, there were obvious differences in the spatial distribution of tourism resources in each township. In this study, the spatial distribution of tourism resources in Longde County was analyzed using the scale index. In addition, the scale of tourism resources in each township in Longde County was calculated and visualized using Formula (1) (Figure 4). The average scale index of tourism resources in Longde County was 1.90, indicating that the resource endowment of Longde County was relatively high, but the scale indexes of different towns were quite different. A high-density core area centered on Chengguan Town was formed, with the highest resource scale index of 4.03; meanwhile, Wenbao Township, which is famous for its cultural history and culture, followed with 2.89. The lowest was Zhangcheng Township (0.91), which was far lower than the high-density core area, and the scale index of eight townships was below the average level. In general, Longde County was rich in tourism resources, but there were large-scale differences at the township level.

#### 4.2.2. Spatial Difference Analysis

There were obvious spatial differences between various tourism resources in Longde County, with an overall variation coefficient of 55.13%. Chengguan Town and Wenbao Township were the two largest towns, with 361 and 237 tourist resources, respectively. Chengguan Town, as the resident town of Longde County government, was the regional political and cultural center. Similarly, part of Wenbao Township was located in Gansu Province, and the continuous exchange and integration of ethnic cultures along the border also determined its rich human tourism resources.

**Figure 5:**
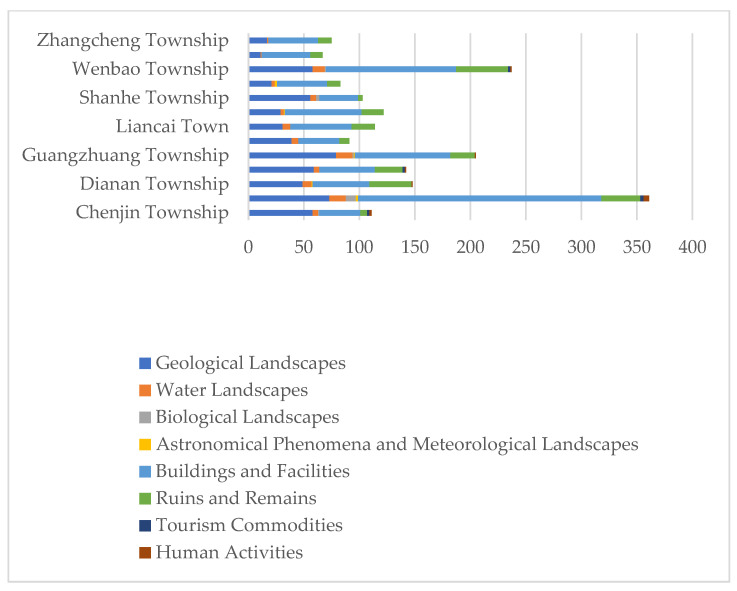
Quantity and structure of tourism resources in each township of Longde County.

In contrast, Yanghe Township and Zhangcheng Township had the least tourism resources. Limited by the complex terrain and poor accessibility in the mountainous area in the north of Longde County, the number of objects of tourism resources was less than 80 and the types were relatively single, mainly comprising natural tourism resources (Figure 5). In each type, the coefficient of variation of biological landscapes, astronomical phenomena and meteorological landscapes, tourism commodities and human activities all exceeded 100% (Table 3), indicating that the spatial differentiation of these tourism resources was significant. The biological landscapes were mainly distributed in Chengguan Town, accounting for 56.25% of the total. Human activities were distributed in Wenbao Township, which was mainly related to the large population in Wenbao Township. In a word, the spatial distribution of tourism resources in Longde County was uneven, especially the biological landscapes, tourism commodities and human activities.

#### 4.2.3. Kernel Density Analysis

**Figure 6:**
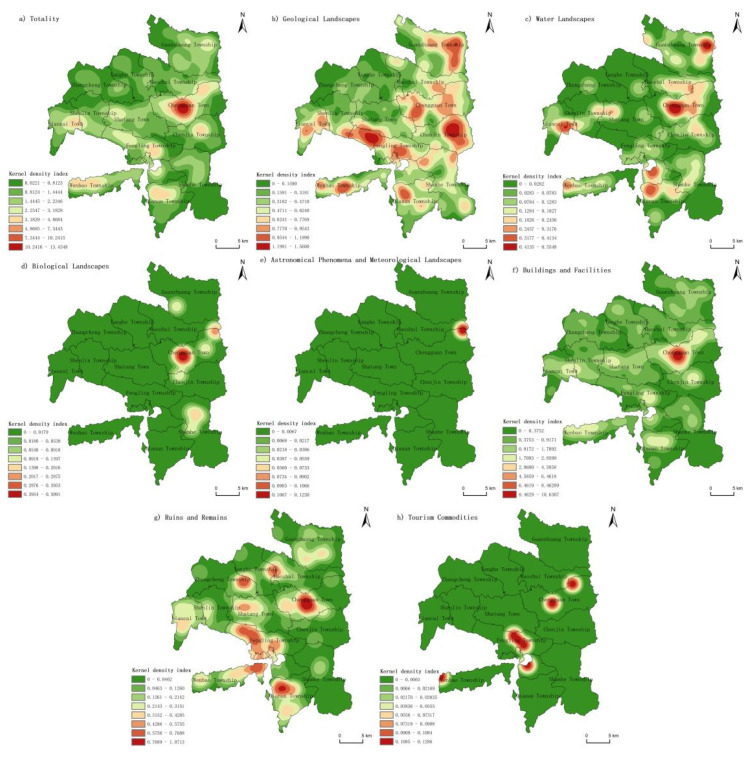
Kernel density of various types of tourism resources in Longde County.

Through a kernel density analysis, this study intuitively described the spatial agglomeration characteristics of tourism resources in Longde County (Figure 6). Considering the mobility and non-entity of human activity tourism resources, we will not discuss them. On the whole, the tourism resources in Longde County were widely and unevenly distributed in space, with a great spatial difference, which was mainly manifested as the “radiation” agglomeration distribution state with Chengguan Town as the core. Chengguan Town was a high-density core area, while Wenbao Township, Dianan Township and Liancai Town were the sub-density core areas. At the same time, the tourism resources in Longde County had a distribution trend of “along the road, around the city and near the water” and the spatial distribution characteristics of “dispersed in large areas and concentrated in small areas”. Most of them were concentrated around the township government, and a small number of them were distributed in areas with complex terrain conditions and poor traffic conditions. Each government station was the political and cultural center of each town, with complete infrastructure, high transportation accessibility, dense population, and a long history and culture, which was conducive to the formation and development of tourism resources, especially the types of buildings and facilities, ruins and remains, and so on. The surrounding areas along traffic and water systems were also the main areas of resource concentration. On the one hand, convenient transportation can improve the accessibility of tourism resources development. On the other hand, transportation lines themselves can be developed into scenic corridors and other characteristic tourism resources. As a type of water landscape tourism resource, the water system itself can also promote the generation and agglomeration of other types of tourism resources around it. As a border town with Gansu Province, Wenbao Township is a multi-ethnic gathering area. Multi-ethnic cultures converge and integrate, which is conducive to the development of cultural tourism resources, and thus, it became a sub-density core area. In contrast, in Zhangcheng Township, Yanghe Township and the southern part of Shanhe Township, limited by the rugged and complex terrain conditions and sparse population density, the number of tourism resources was small, showing irregular discrete distribution, and the kernel density index was only between 0.0221 and 0.8123 (Figure 6a).

From the main types of tourism resources, the geological landscapes tourism resources were widely distributed, showing the characteristics of multi-core spatial distribution. As the eastern part of Chenjin Township, Guanzhuang Township and Chengguan Town were adjacent to Liupan Mountain, geological landscape tourism resources were rich. The core density in the center of the three regions reached 1.5600, which was much higher than that in other regions (Figure 6b). In terms of space, the water landscapes mainly showed obvious “water system dependency”, which was distributed around the major water systems, such as Haoshuichuan River and Ganweizi River, in Longde County. Therefore, Chengguan Town, Haoshui Township, Wenbao Township and other areas had a large number of water landscapes, generally showing irregular banded distribution (Figure 6c). The kernel density distributions of biological landscapes as well as astronomical phenomena and meteorological landscapes are shown in Figure 6d,e, respectively. The above two kinds of tourism resources in Longde County were small in quantity and mainly showed a mononuclear distribution in space. The biological landscapes were mainly distributed in Chengguan Town, while the astronomical phenomena and meteorological landscapes were mainly distributed in the border area between Chengguan Town and Haoshui Township, with kernel density indexes of 0.5091 and 0.1230, respectively. Buildings and facilities tourism resources were scattered in the county, but the core areas and sub-core areas were particularly prominent. As the buildings and facilities tourism resources in Longde County constituted the main part of the whole county, their spatial pattern was generally similar to the total tourism resources. In terms of space, the tourism resources of buildings and facilities showed a radial distribution, with Chengguan Town as the core area and the density of the core reached 10.6307 (Figure 6f). This was mainly related to Chengguan Town as the political, economic and cultural center of the county. Ruins and remains tourism resources were connected in the southwest of Longde County. Besides Chengguan Town, the high-density core areas also included Shatang Town, Fengling Township and Dianan Township (Figure 6g). Frequent prehistoric human activities gave the southwestern region of Longde County the innate conditions to make use of the ruins as a tourism resource. A large number of neolithic sites were unearthed, forming the high-density core area of the tourism resources of ruins and remains. Tourism commodities showed an obvious dot distribution in space, including Chengguan Town, Wenbao Township and Fengling Township, mainly because the above areas had a high population density and good tourism market potential (Figure 6h).

## 5. Discussion

### 5.1. The Physical Geographical Environment Factors That Influenced the Spatial Pattern

**Figure 7:**
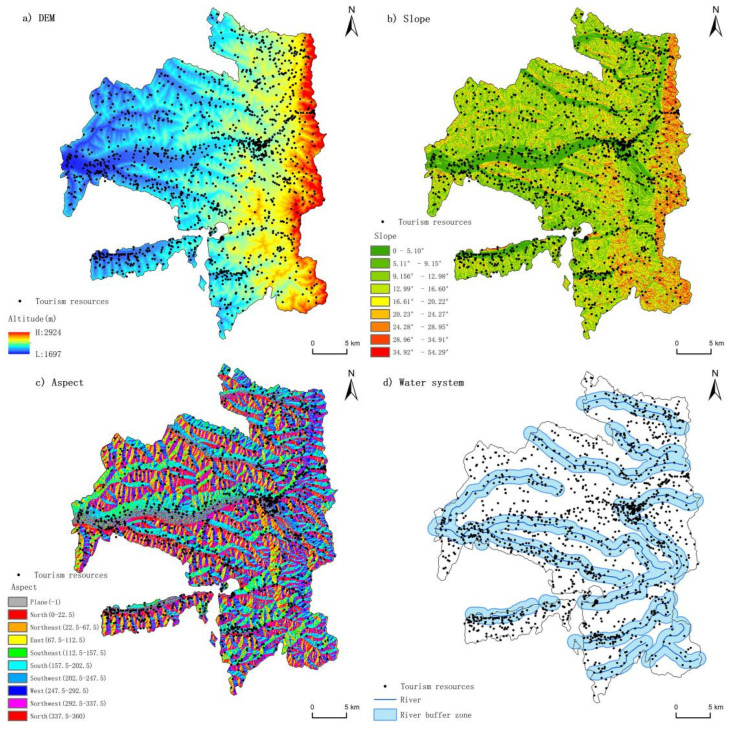
The relationship between the distribution of tourism resources and the natural geographical environment in Longde County. Data source: National Basic Geographic Information System database and the National Basic Geographic Information System database.

The regional natural geographical environment was one of the preconditions that affected the spatial pattern of tourism resources and it played an important role in the development of tourism resources. Through the surface analysis of the landform and the analysis of the river buffer zone in Longde County, we found that the distribution of tourism resources was related to the natural environment (Figure 7a–d).

Because Longde County belongs to the Loess Plateau area with high terrain, its tourism resources were located at a higher altitude; most of them were distributed in regions above 1700 m. Within the range of 1800–2200 m, the number of tourism resources increased sharply and reached an extreme value in the range of 2000–2200 m. There were 620 tourism resources in this range, accounting for 30.26% of the total. In the range from 2000 m to 2200 m, there were abundant geomorphic types, mainly the loess hilly and gully region with flat terrain and deep soil layer, creating many unique geomorphic landscapes. When the altitude exceeded 2200 m, the landform type was mainly wet soil and stone mountainous area, the landscape value was relatively reduced and the transportation accessibility of resources was low, which led to a sharp decline in the number of tourism resources in this altitude range. From the perspective of the slope, there were 1072 tourism resources with a slope less than 10°, accounting for about 64.35% of the total. Approximately 90.93% of the object tourism resources were distributed in the area with a slope of less than 20°. The smaller slope was conducive to infrastructure construction and cultural activities, thus generating and developing more tourism resources. Regarding the slope aspect, tourism resources were distributed in all aspects, among which the plane was the least, with only one, accounting for 0.06% of the total tourism resources. Among the other aspects, the distribution of tourism resources was relatively balanced, with small spatial differences, among which the southern, western and southwestern distribution of tourism resources was greater, accounting for 55.10% of the total.

The water system was also an important natural factor that affected the distribution of tourism resources, especially the water landscape tourism resources. Longde County had Haoshuichuan River, Yu River, Ganweizi River, Luojiaxia and other water systems, as well as Sanlidian Reservoir, Longde Reservoir and other water resources, becoming an important location for water landscapes tourism resources. In this study, 1 km was taken as the buffer distance for the water system of Longde County (Figure 7d). According to statistics, a total of 1184 resources were in the 1 km buffer, accounting for 57.78% of the total resources. Among them, there were 76 water landscapes tourism resources, accounting for 75.24% of the total number of similar resources, while the other types of tourism resources were less dependent on the water system. Water resources, such as rivers and reservoirs, were not only tourism resources themselves but also the natural environment that the tourism resources relied on.

### 5.2. The Social and Environmental Factors That Influenced the Spatial Pattern

**Figure 8:**
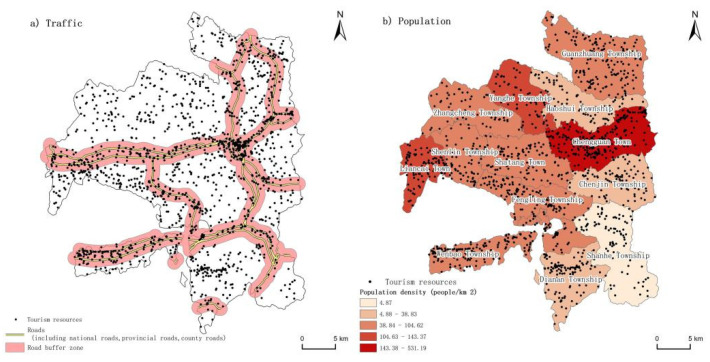
The relationship between the distribution of tourism resources and the social environment. Data source: the Statistical Bulletin of National Economic and Social Development and the National Basic Geographic Information System database.

Regional transportation accessibility was also an important factor that affected the distribution and development of tourism resources. Convenient transportation could greatly improve the accessibility of tourism resources and facilitate the development of tourism resources. In this study, the buffer analysis method was used to build a buffer zone of 1 km distance with the transportation lines (national roads, provincial roads, county roads, etc.) in Longde County as the benchmark (Figure 8a), and we used ArcGIS to calculate the number of tourism resources contained in the buffer zone. The results showed that there were 793 resources in the 1 km buffer zone, accounting for 38.70% of the total. Compared with county roads and national roads, tourism resources were more common in the provincial roads buffer zone. Similarly, the number of tourism resources within each different buffer distance was counted, and it was found that the number of tourism resources decreased with the increase in distance from the transportation lines. For example, Chengguan Town was located in the center of the transportation line with a dense transportation network and high transportation accessibility. It had a large number of tourism resources, 355 in total, accounting for 17.33% of the total resources. In contrast, Yanghe Township was far away from all the transportation lines, and the number of tourism resources was small, with a total of 67, accounting for only 3.27% of the total resources. In conclusion, the accessibility of transportation was one of the important reasons that affected the distribution and development of tourism resources in this kind of region.

In addition, there was a certain relationship between the population density and the spatial pattern of tourism resources. Based on the Seventh National Census Bulletin data of Longde County in 2021, this study calculated the population density of each township in Longde County with the township as the research scale (Figure 8b). It can be seen from the figure that the population distribution of Longde County was concentrated in the middle area, and the population densities in the northern and southern areas were relatively low. Compared with Figure 4, the statistical analysis showed that there was a positive correlation between the population and tourism resources, where the number of tourism resources was also higher in areas with a large population, while the number of tourism resources was lower in areas with a sparse population. For example, Chengguan Town had a relatively high population density and a high number of resources. It had 361 tourism resources, including 260 humanistic tourism resources, accounting for 72.02% of the total resources of the town. This was closely related to the frequent human activities, which promoted the formation of a variety of human tourism resources. In contrast, Haoshui Township and Zhangcheng Township had smaller population densities and a smaller number of tourism resources, with only 46 human tourism resources. Therefore, the population density was also an important factor that affected the spatial pattern of tourism resources, especially human tourism resources.

### 5.3. Analysis of the Influencing Factors Based on a Multiple Linear Regression Model

**(1)**  **Selection of the explanatory variables and sample data preprocessing**

The analysis results discussed in Section 5.1 and Section 5.2 showed that the spatial pattern of tourism resources was comprehensively affected by the topography, water system, transportation, population density and other factors. Therefore, this study selected 1666 effective tourism resources as sample data and constructed a multiple linear regression model in terms of two factors, namely, the natural environment and social environment, to quantitatively analyze the influence of the spatial pattern of tourism resources in Longde County. At the same time, combined with relevant literature research results, in accordance with the principles of science, operability and comparability, this study preliminarily selected one explained variable and seven explanatory variables (Table 4).

The preprocessing of variable data was realized using ArcGIS 10.6 software. Among them, the kernel density value of tourism resources *Y*: First, the raster data of the kernel density of tourism resources in Longde County were obtained using kernel density analysis. Then, the kernel density value of the resource sample points was obtained as the explained variable by tool-adding the surface information. Altitude *X*_1_: the altitude of the resource sample points was obtained as the explanatory variable by adding surface information through the tool. Slope *X*_2_ and aspect *X*_3_: based on the DEM data, the tool surface analysis slope (aspect) was used to obtain the raster data of the slope (aspect) of tourism resources, and the slope and aspect values of resource sample points were taken as explanatory variables by adding the surface data. Distance from the transportation line *X*_4_ and distance from the water system *X*_5_: through the nearest-neighbor analysis in the neighborhood analysis tool, the nearest distance of each resource sample point to the main transportation lines and water system was calculated as the explanatory variable. Population density *X*_6_: in this study, the population density data obtained in Section 5.2 was rasterized and the population density value of the location of each resource point was calculated as the explanatory variable by adding the surface information tool. Distance from the main administrative unit *X*_7_: In this study, the county government of Longde County was chosen as the main administrative unit. As the political, economic and cultural center of Longde County, it not only had a superior natural environment, but also a dense transportation network, frequent human activities, and a rich collection of tourism resources. Through the point distance analysis tool, the distance between each resource sample point and the county government resident in Longde County was calculated as the explanatory variable.

**(2)**  **Model building and data analysis**

Combined with the corresponding indicators, a multiple linear regression model was established with the kernel density value of tourism resources as the explained variable, and the altitude, slope, aspect, distance from the water system, distance from the transportation lines, population density and distance from the main administrative units as the explanatory variables. The linear model is shown in Equation (5).

In this study, SPSS software was used to conduct a multiple linear regression analysis on the relevant data, and the results are shown in Table 5 and Table 6. The Durbin–Watson coefficient was 1.723, indicating that the criterion for independence between samples was satisfied. R^2^ was 0.564 and the adjusted R^2^ was 0.562, indicating that this model fit the data well (Table 5).

Table 6 is the regression coefficient table, which mainly shows the regression coefficient and significance test of each explanatory variable in the regression model. The table shows that the regression coefficients of the seven independent variables in the model were −0.003, −0.024, 0.001, −0.000132, −0.001, 0.008 and −0.000082. The t values of the significance test of the regression coefficient were 14.864, −10.146, −3.056, 1.421, −5.258, −15.018, 24.901 and −9.216. Except for the aspect *X*_3_, the other explanatory variables were all significant at the level of 0.01, indicating that they had a significant impact on the spatial distribution of tourism resources. Among them, the coefficient of population density *X*_6_ was positive, indicating that the greater the population density, the more intensive the tourism resources, while the influence of other explanatory variables on the dependent variable was negative, indicating that with the increase in the value of explanatory variables (*X*_1_, *X*_2_, *X*_4_, *X*_5_, *X*_7_), the tourism resources in this region tended to be dispersed. The aspect *X*_3_ did not pass the significance test because the tourism resources in Longde County were distributed in different aspects, the quantity was average and the difference was small.

Table 7 shows the collinearity diagnosis of the explanatory variables. From Table 6, the eigenvalues of the six variables (excluding the aspect *X*_3_) were 5.961, 0.784, 0.324, 0.271, 0.157 and 0.069 for *X*_1_, *X*_2_, *X*_4_, *X*_5_, *X*_6_ and *X*_7_, respectively. The conditional index values were all lower than 10. In addition, it could be seen that the tolerance of all explanatory variables was not less than 0.1 and VIF was not higher than 10. A comprehensive analysis inferred that there was no collinearity problem among the explanatory variables in this model.

Based on the above analysis results, altitude (*X*_1_), slope (*X*_2_), distance from a transportation line (*X*_4_), distance from a water system (*X*_5_), population density (*X*_6_), and distance from a main administrative unit (*X*_7_) had significant impacts on the spatial pattern of tourism resources. In this model, the collinearity among all explanatory variables was weak, the independence between samples was satisfied and the model fit degree was good, indicating that the data analysis results were valid and the model was reliable. The regression coefficients of explanatory variables obtained in Table 4 were substituted into Equation (5) to obtain the regression Equation (6):

Y = 9.915 − 0.003*X*_1_ − 0.024*X*_2_ − 0.000132*X*_4_ − 0.001*X*_5_ + 0.008*X*_6_ − 0.000082*X*_7_ (6)

According to Formula (6), when all explanatory variables were 0, the tourism resource density was high, which was 9.915, which verified the conclusion that Longde County had rich tourism resources. Altitude, slope, distance from a transportation line, distance from a water system, and distance from a main administrative unit were inversely proportional to the kernel density of tourism resources, indicating that tourism resources tended to be distributed discretely when the values of explanatory variables, such as elevation and distance from a water system, increased continuously. In contrast, the coefficient of population density was 0.008, showing that the place with population agglomeration had more intensive tourism resources. By comparing the coefficients of all explanatory variables, it can be seen that the slope was the most significant factor that affected the spatial pattern of tourism resources, with a coefficient of −0.024, followed by population density, altitude, distance from a water system, distance from a transportation line and distance from a main administrative unit. The regression results showed that the tourism resources in Longde County tended to be distributed in areas with a low altitude, gentle terrain and dense population. However, in remote areas (including far away from transportation lines, water systems and main administrative units), the distribution of tourism resources was lower and the density was relatively low.

## 6. Conclusions

In this study, the scale index, kernel density analysis, spatial difference and other research methods were used to analyze the mathematical characteristics and spatial pattern characteristics of the tourism resources in Longde County. Furthermore, this study constructed a multiple linear regression model to quantitatively analyze the factors that affected the spatial pattern of tourism resources in Longde County. Finally, by combining with the local tourism development policy, this study put forward the optimization path of the spatial pattern of tourism resources. We drew the following three main conclusions: 

(1) The Longde County tourism resources database contained a total of 2049 tourism resources, covering 8 main types, 20 sub-types, and 70 fundamental types in the National Standard for the “Classification, investigation and evaluation of tourism resources” (GB/T18972-2017), accounting for 100%, 86.96% and 63.63% of the national standard, respectively. Cultural tourism resources were more abundant, where the number was about twice as many as the natural tourism resources. In the main type, the number of buildings and facilities was the largest, with a total of 970. The cultural landscape complex was the most abundant sub-type, and the hill landscape was the most abundant among the basic types, accounting for about 27.33% of the total resources. In addition, the hierarchical structure of the tourism resources in Longde County was arranged in a “pyramid” shape. The higher the quality of resources, the lower the quantity. Among them, only 16 grade-5 tourism resources were found, accounting for the lowest proportion at approximately 0.63% of the total resources. There were 1586 ordinary tourism resources, which constituted the main body of tourism resources in Longde County.

(2) The spatial distribution of tourism resources in Longde County was extremely uneven and the tourism resources showed an agglomeration distribution. First of all, the scale of tourism resources varied greatly between townships. Chengguan Town was the most abundant, with a resource scale index of 4.03, while Zhangcheng Township’s scale index was only 0.91. Second, the spatial differences of all types of tourism resources were significant, and the coefficients of variation of astronomical phenomena and meteorological landscapes, biological landscapes, human activities and tourism commodities were all higher than 100%. Finally, from the results of the analysis of the kernel density of tourism resources, the tourism resources in Longde County were mainly manifested as a “radiation” agglomeration distribution state with Chengguan Town as the core. Chengguan Town was the high-density core area, while Wenbao Township, Dianan Township and Liancai Town were the sub-density core areas. At the same time, the tourism resources in Longde County had the distribution trend of “along the road, around the city and near the water” and the spatial distribution characteristics of “dispersed in large areas and concentrated in small areas”. The spatial pattern of tourism resources of different main types was different. The natural factors and cultural conditions of different regions had created the spatial differentiation of different types of tourism resources in Longde County.

(3) Altitude, slope, distance from a transportation line, distance from a water system, population density and distance from a main administrative unit were important factors that affected the spatial pattern of tourism resources. Among them, the terrain slope was the most significant factor that affected the spatial pattern of tourism resources, followed by population density, altitude, distance from a water system, distance from a transportation line and distance from a main administrative unit. With the continuous rise in altitude, the number of resources first increased and then decreased sharply, mainly distributed in the altitude range of 2000–2200 m. The regression results showed that the tourism resources in Longde County were more distributed in the areas with a low altitude, gentle terrain and dense population. However, the number of objects of tourism resources in areas far away from transportation lines, water systems and main administrative units was small, showing a discrete distribution.

Based on the above results, three suggestions were proposed for the sustainable development of tourism in Longde County. First, the tourism resources in Longde County had an obviously unbalanced distribution in space. The local government should give full play to the advantages of intangible cultural remains and create characteristic cultural tourism products that cover the whole area of Longde County. At the same time, the government should combine the cultural accumulation and historical and cultural resources of ancient counties to build cultural landscapes, such as Longde County Cultural and Museum, cultural exchange square and characteristic historical sites, and vigorously carry out Qinqiang opera performances, Ma Shehuo and other cultural activities. Second, the government should actively promote the development of protective tourism activities, such as ecotourism to improve the development level of tourism while protecting the ecological environment to promote the sustainable development of tourism. Third, the government should improve the construction of tourism infrastructure and improve tourism accessibility. It is very wise to increase highway mileage reasonably, improve tourism efficiency by using the “Temporal compression effect” and promote the development of tourism in a virtuous circle. Finally, under the promotion of various national policies, the local government should take the sustainable development of regional tourism as the purpose, reasonably control the exploitation of tourism resources, and balance the relationship between regional tourism development and the local economy, society and ecological environment.

Due to the lack of relevant data and the difficulty in obtaining some data, the transportation line data involved in this study were mainly national roads, provincial roads and county roads. The relevant data for other types of roads were lacking and were not analyzed. The index system of influencing factors still needs to be improved, and subsequent research could rely on various road data and finer water system data to further improve the scientific nature of the related analysis. Finally, it is necessary to reflect on whether the current tourism development mode is conducive to sustainable development and what scope should be controlled for the exploitation of tourism resources. Future research should focus on the sustainability of tourism resources exploitation and tourism development and conduct quantitative research to determine to what extent regional tourism development is sustainable.

## Figures and Tables

**Table 1:** Quantity and basic types of tourism resources in Longde County.

Main Types			Subtypes		Number of Fundamental Types
Types	Number	Proportion (%)	Types	Number	
**A** Geological landscapes	602	29.38	**AA** Natural landscape complex	583	3
			**AB** Geological and tectonic features	6	2
			**AC** Surface morphology	13	3
**B** Water landscapes	101	4.93	**BA** Rivers	25	2
			**BB** Lakes	49	3
			**BC** Groundwater	26	2
			**BD** Ice and snow	1	1
**C** Biological landscapes	16	0.78	**CA** Vegetation landscape	14	3
			**CB** Wildlife habitat	2	1
**D** Astronomical phenomena and meteorological landscapes	7	0.34	**DA** Astronomical landscape	1	1
			**DB** Weather and climate phenomena	6	2
**E** Buildings and facilities	970	47.34	**EA** Cultural landscape complex	509	9
			**EB** Practical buildings and facilities	360	13
			**EC** Landscape architecture	101	10
**F** Ruins and remains	290	14.15	**FA** Material cultural remains	269	1
			**FB** Intangible cultural remains	21	4
**G** Tourism commodities	45	2.20	**GA** Agricultural products	33	2
			**GC** Handmade crafts	12	4
**H** Human activities	18	0.89	**HA** Personnel activity record	12	2
			**HB** Festivals	6	2
Sum	2049	100.00	20	2049	70

Data source: Longde County tourism resource database.

**Table 2:** Grade distribution of tourism resources in Longde County.

Grade	Excellent Tourism Resources			Ordinary Tourism Resources		Sum
	5	4	3	2	1	
Number	16	47	400	505	1081	2049
Proportion (%)	0.78	2.29	19.52	24.65	52.76	100.00

**Table 3:** Quantity and variation coefficient of tourism resources in Longde County.

Main Types	Geological Landscapes	Water Landscapes	Biological Landscapes	Astronomical Phenomena and Meteorological Landscapes	Buildings and Facilities	Ruins and Remains	Tourism Commodities	Human Activities	Total
Number	602	101	16	7	970	290	45	18	2049
CV (%)	49.69				65.98				55.13
	46.57	68.30	209.13	138.51	71.27	62.91	153.96	177.76	

**Table 4:** Variable and variable description.

	Variable	Variable Description
Explained variable	*Y*	Kernel density value of tourism resources
Explanatory variables	*X*_1_	Altitude (m)
	*X*_2_	Slope (°)
	*X*_3_	Aspect (%)
	*X*_4_	Distance from a line of transportation (m)
	*X*_5_	Distance from a water system (m)
	*X*_6_	Population density (people/km^2^)
	*X*_7_	Distance from a main administrative unit (m)

Data source: the Data Center for Resources and Environmental Sciences, Chinese Academy of Sciences, the National Basic Geographic Information System database, the Statistical Bulletin of National Economic and Social Development, and the National Basic Geographic Information System database.

**Table 5:** Model summary.

Model	R	R^2^	Adjusted R^2^	Error of the Standard Estimate	Durbin–Watson
1	0.751	0.564	0.562	1.87507611610345	1.723

**Table 6:** Model regression coefficient.

Model	Unstandardized Coefficient		Standard Coefficient	t	Sig.	Collinearity Statistic	
	B	Standard Error	Trial Version			Tolerance	VIF
Constant	9.915	0.667		14.864	0.000		
Altitude *X*_1_ (m)	−0.003	0.000	−0.211	−10.146	0.000	0.605	1.652
Slope *X*_2_ (°)	−0.024	0.008	−0.055	−3.056	0.002	0.815	1.227
Aspect *X*_3_ (%)	0.001	0.001	0.023	1.421	0.155	0.985	1.015
Distance from a line of transportation *X*_4_ (m)	0.000	0.000	−0.094	−5.258	0.000	0.827	1.209
Distance from a water system *X*_5_ (m)	−0.001	0.000	−0.259	−15.018	0.000	0.881	1.136
Population density *X*_6_ (people/km^2^)	0.008	0.000	0.527	24.901	0.000	0.587	1.705
Distance from a main administrative unit *X*_7_ (m)	−8.161 × 10^−5^	0.000	−0.218	−9.216	0.000	0.471	2.123

**Table 7:** Collinearity diagnosis.

Model	Dimension	Eigenvalue	Condition Index	Variance Ratio							
				Constant	*X*_1_ Altitude	*X*_2_ Slope	*X*_3_ Aspect	*X*_4_ Distance from a Line of Transportation	*X*_5_ Distance from a Water System	*X*_6_ Population Density	*X*_7_ Distance from a Main Administrative Unit
1	1	5.961	1.000	0.00	0.00	0.01	0.00	0.01	0.01	0.00	0.00
	2	0.784	2.757	0.00	0.00	0.00	0.00	0.18	0.00	0.22	0.02
	3	0.432	3.714	0.00	0.00	0.02	0.03	0.15	0.44	0.06	0.04
	4	0.324	4.291	0.00	0.00	0.05	0.01	0.61	0.41	0.08	0.02
	5	0.271	4.691	0.00	0.00	0.69	0.03	0.03	0.05	0.00	0.06
	6	0.157	6.168	0.00	0.00	0.00	0.65	0.01	0.08	0.23	0.19
	7	0.069	9.328	0.02	0.03	0.11	0.26	0.03	0.00	0.38	0.31
	8	0.003	47.742	0.98	0.97	0.12	0.00	0.00	0.00	0.04	0.37

Explained variable: Y, kernel density value.

## Data Availability

Not applicable.
